# Supplementary material for: Asserting a Functional Neurological Symptom Disorder with a Complementary Diagnostic Approach: A Brief Report
Source: Children (Basel). 2023 Sep 25;10(10):1601. doi: 10.3390/children10101601 (PMC10605693; doi:10.3390/children10101601)
Supplement: Supplementary file 1 [file children-10-01601-s001.zip › children-2588767-supplementary.pdf]

Supplementary Table S1: Clinical, biological and radiological assessments. There were no anomalies in the tests carried out.

	Explorations performed
**Blood**	Hemogram, ionogram, inflamatory markers<br>Viral PCR and RT-PCR for enterovirus, influenza virus, measles virus, mumps virus, Epstein-Barr Virus, cytomegalovirus, human herpes virus 6 and 8, HIV<br>Beta-D-glucan<br>Interferon activity and Simoa digital ELISA.<br>Chromatography of blood organic amino acids, search for enzymatic deficiency, pyruvate / lactate cycle, glycosaminoglycans, acylcarnitine<br>Anti-nuclear antibody, anti-DNA, Anti Aquaporin 4 and MOG<br>Toxic cause (copper, lead, vitamin, exogenous)<br>Lymphocyte immunophenotyping and complement dosage
**CSF**	Proteinorachia, glycorachia, leucocyte and cell count. Bacterial and fungal culture<br>Viral PCR and RT-PCR for enterovirus, cytomegalovirus, human herpes simplex virus 1 and 2<br>Interferon activity and Simoa digital ELISA.<br>Autoimmune encephalitis antibody panel including MOG, Aquaporin 4, CASPR2, anti NMDAr and GAD
**Urine**	Chromatography of urinary organic amino acids
**Electroencephalograms**	4 were recorded including during sleep and long duration with video recording but no seizures or abnormalities of the tracing were recorded.
**Radiological survey**	Brain CT scanner, Brain and spinal cord MRI
**Ophtalmological work-up**	visual acuity, pupils, side vison, visual field, eye movement, eye pressure, retina and fundus
**Otorhinolaryngologist work-up**	nasofibroscopy, auditory tests
